# Identification of New Protein Interactions between Dengue Fever Virus and Its Hosts, Human and Mosquito

**DOI:** 10.1371/journal.pone.0053535

**Published:** 2013-01-11

**Authors:** Dumrong Mairiang, Huamei Zhang, Ann Sodja, Thilakam Murali, Prapat Suriyaphol, Prida Malasit, Thawornchai Limjindaporn, Russell L. Finley

**Affiliations:** 1 Center for Molecular Medicine and Genetics, Wayne State University School of Medicine, Detroit, Michigan, United States of America; 2 Department of Biology, Wayne State University, Detroit, Michigan, United States of America; 3 Bioinformatics and Data Management for Research Unit, Faculty of Medicine Siriraj Hospital, and Center for Emerging and Neglected Infectious Diseases, Mahidol University, Bangkok, Thailand; 4 Dengue Hemorrhagic Fever Research Unit, Office for Research and Development, Faculty of Medicine Siriraj Hospital, Mahidol University, Bangkok, Thailand; 5 Medical Biotechnology Research Unit, National Center for Genetic Engineering and Biotechnology, National Science and Technology Development Agency, Bangkok, Thailand; 6 Department of Anatomy, Faculty of Medicine Siriraj Hospital, Mahidol University, Bangkok, Thailand; 7 Department of Biochemistry and Molecular Biology, Wayne State University School of Medicine, Detroit, Michigan, United States of America; Johns Hopkins University, Bloomberg School of Public Health, United States of America

## Abstract

The four divergent serotypes of dengue virus are the causative agents of dengue fever, dengue hemorrhagic fever and dengue shock syndrome. About two-fifths of the world's population live in areas where dengue is prevalent, and thousands of deaths are caused by the viruses every year. Dengue virus is transmitted from one person to another primarily by the yellow fever mosquito, *Aedes aegypti*. Recent studies have begun to define how the dengue viral proteins interact with host proteins to mediate viral replication and pathogenesis. A combined analysis of these studies, however, suggests that many virus-host protein interactions remain to be identified, especially for the mosquito host. In this study, we used high-throughput yeast two-hybrid screening to identify mosquito and human proteins that physically interact with dengue proteins. We tested each identified host protein against the proteins from all four serotypes of dengue to identify interactions that are conserved across serotypes. We further confirmed many of the interactions using co-affinity purification assays. As in other large-scale screens, we identified some previously detected interactions and many new ones, moving us closer to a complete host – dengue protein interactome. To help summarize and prioritize the data for further study, we combined our interactions with other published data and identified a subset of the host-dengue interactions that are now supported by multiple forms of evidence. These data should be useful for understanding the interplay between dengue and its hosts and may provide candidates for drug targets and vector control strategies.

## Introduction

Infection with one of the four serotypes of dengue virus can result in dengue fever and the life-threatening sequalae, dengue hemorrhagic fever and dengue shock syndrome [1]. Dengue infects as many as 50 million people each year, resulting in thousands of deaths, especially in children [2]. Currently, there is no effective antiviral drug available and intense efforts to develop a vaccine are still ongoing [2]–[4]. About 2.5 billion people around the world live in areas where there is a significant risk of dengue infection and recently the virus has expanded into areas where it previously did not exist or had been eradicated [5], [6]. Dengue is an arthropod-borne virus (arbovirus) that is transmitted by mosquito vectors, primarily the urban-dwelling species *Aedes aegypti* [1]. Mosquitoes become infected by taking a blood meal from an infected human, and after an incubation period of 7–14 days, during which the virus disseminates from the midgut and proliferates in salivary glands, the mosquito is capable of infecting another person [1], [7], [8]. Because of the essential role of the mosquito in the virus transmission cycle, a number of efforts to combat dengue have focused on vector control strategies [1], [9]–[13]. A better understanding of how dengue interacts with its mosquito host to allow replication and dissemination would be helpful in the development of these strategies.

**Figure 1:**
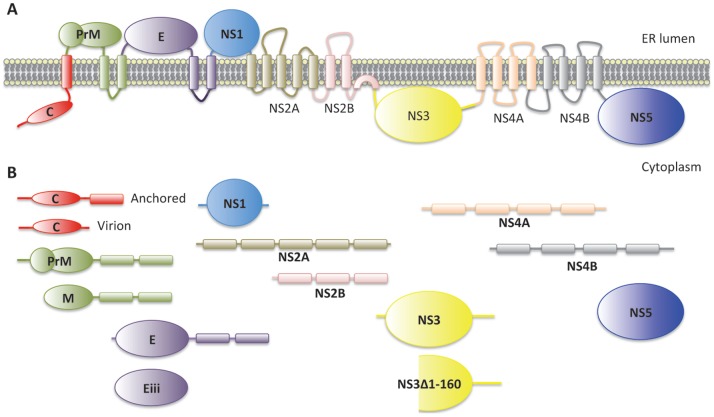
Dengue virus proteins. (A) The dengue polyprotein in the ER membrane prior to processing. (B) The coding regions for the fourteen dengue virus proteins and partial peptides shown were separately cloned into yeast two-hybrid plasmids.

Dengue belongs to the *Flaviviridae* family, which includes the West Nile and Yellow fever viruses [14]. There are four distinct serotypes of dengue virus (DENV1–4), which are as different from each other as are other separately classified viruses, such as the West Nile and Japanese Encephalitis viruses [15], [16]. Here we will refer to all serotypes generically as dengue virus unless we are talking about a specific serotype. The dengue viral genomes consist of a positive single-stranded RNA encoding ten proteins; capsid (C), membrane protein (M), envelope protein (E), and seven nonstructural proteins, NS1, NS2A, NS2B, NS3, NS4A, NS4B and NS5 [17]. These proteins are translated as a polyprotein (Figure 1A), which is cleaved into individual proteins during maturation by the host proteases furin and signalase on the lumenal side of the ER, and by the dengue NS2B/NS3 protease complex on the cytoplasmic side [17], [18]. A mature virion contains a molecule of viral RNA encapsulated in capsid and enveloped by a membrane that contains M and E proteins [17]. NS5 is a large multifunctional protein with a C-terminal RNA-dependent-RNA polymerase domain that is required for viral replication [19] and an N-terminal methyltransferase domain required for RNA capping [20]. The functions of the remaining nonstructural proteins, NS1, NS2A, NS4A and NS4B, are less well understood. A number of functional studies, however, have shown that these proteins are involved in dengue pathogenesis and immune response in humans. Individual expression of NS2A, NS4A, or NS4B proteins, for example, can enhance replication of an interferon (IFN)-sensitive virus and down-regulate the expression of IFN-β-stimulated reporter genes, suggesting that these proteins contribute to inhibition of the IFN-mediated viral defense system [21]. NS5 also inhibits expression of IFN-stimulated genes [22]. NS1, which is a secreted protein, contributes to immune evasion at least in part by interfering with the complement system [23], [24]. Although viral replication and maturation occur in the cytoplasm, endoplasmic reticulum, and Golgi apparatus [17], the capsid and NS5 proteins are also detected in the nucleus [25]–[27]. The potential nuclear roles of these proteins are poorly understood [28], [29].

A more detailed picture of how dengue virus interacts with its hosts has begun to emerge from studies aimed at identifying interactions between dengue proteins and host proteins. Several studies have focused on specific genes or pathways that relate to dengue pathogenesis. For example, an interaction between dengue capsid and human Death-domain Associated protein (DAXX) was identified and shown to increase Fas-mediated and CD137-meditated apoptosis of liver cells [25], [30], [31]. In another example, NS5 was found to bind either directly or indirectly to the critical IFN signaling pathway transcription factor, Signal Transducer and Activator of Transcription 2 (STAT2) leading to STAT2 degradation [22], [32]. Meanwhile, several recent large-scale studies have identified novel dengue-host protein interactions. Khadka et al., used yeast two-hybrid screens to identify 139 protein interactions involving 105 human liver proteins and fragments of eight dengue serotype 2 proteins [33]. They went on to show that RNAi-mediated knock down of a few of these human proteins inhibited a dengue replicon suggesting that these host factors may be important for the dengue life cycle. In another study, Le Breton et al., used yeast two-hybrid screens to identify interactions involving 108 human proteins and the NS3 and NS5 proteins from dengue serotype 2 and several other flaviviruses [34]. In yet another study, a bacterial two-hybrid screen was used to detect 31 dengue-interacting human proteins [35]. To date, however, no large-scale two-hybrid screens have been reported to detect dengue-mosquito protein interactions. In the largest study reported thus far for mosquito proteins, Colpitts et al., used tandem affinity purification assays in an *Aedes albopictus* cell line to identify protein interactions involving 19 mosquito proteins and four dengue proteins including capsid, envelope protein, NS2A, and NS4B [36]. Several of the mosquito proteins were histones and in a follow-up study the same group reported that dengue capsid can interfere with nucleosome assembly in human liver cells [29]. Computational approaches have also been used to predict dengue-host protein interactions. Doolittle et al., for example, predicted 4,376 human-dengue and 176 mosquito-dengue protein interactions based on structural similarity between dengue proteins and host proteins [37]. In another study a number of interactions were predicted between mosquito and dengue proteins or RNA based on potential conservation of host-flavivirus interactions [38].

Despite the valuable insights that can be derived from protein interactions, the available data suggest that the dengue-host interactome is still noisy and incomplete. Protein interaction data from high throughput screens and even from literature-curated databases can include many false positive interactions that have no biological relevance [39]–[41]. One way to address this problem is to focus on interactions detected by multiple independent experiments because those interactions are more likely to be biologically relevant [42]–[50]. However, very few dengue-host protein interactions have been identified in more than one experiment. For example, the large-scale two-hybrid screens [33], [34] detected only one interaction in common (between NS5 and human Matrin 3) and only one previously published interaction (between NS5 and human STAT2 [22], [32]). Colpitts et al., identified only one mosquito-dengue protein interaction, between NS2A and myelin protein expression factor (AAEL003670) [36], that was also predicted by Doolittle et al [37]. The lack of overlap between different large-scale datasets is likely due in part to false positives that are detected in one screen but not another. However, even most of the interactions that have been confirmed and functionally validated were detected in only one study, suggesting that the screens also have high false negative rates. Thus, additional large-scale screens may be used to identify new protein interactions and to provide additional evidence for previously identified interactions. In this study, we used yeast two-hybrid screens to identify interactions between proteins from dengue virus and its hosts, mosquito and human. We independently tested most of the interactions by co-purification from insect cells. Furthermore, we tested interactions of host proteins against all four serotypes of dengue virus to identify either serotype-specific or serotype-independent interactions. Our data help define a more complete dengue-host interactome. When combined with the data from previous studies, the data presented here provide a higher confidence set of dengue-host protein interactions supported by multiple forms of evidence.

## Results and Discussion

### Intraviral protein-protein interactions

To identify interactions with dengue proteins we subcloned open reading frames (ORFs) from dengue virus serotype 2 (strain 16681) into the yeast two-hybrid bait vector for expression of the proteins with an N-terminal LexA DNA binding domain (DBD). We constructed a total of 14 baits (Figure 1B). These included baits for all ten full-length dengue proteins: nascent capsid protein (C), precursor of membrane protein (PrM), E, NS1, NS2A, NS2B, NS3, NS4A, NS4B and NS5. These ten proteins are individually cleaved from the viral polypeptide during maturation [17], [51], [52]. We also constructed baits for mature capsid protein (CV) [53], mature membrane protein (M) [54], domain III of envelope protein (Eiii), and a fragment of NS3 lacking the N-terminal 160 amino acids (NS3Δ1–160) (Figure 1B). We subcloned the same 14 dengue ORFs into the yeast two-hybrid activation domain (AD) vector. This enabled us to test for interactions among the 14 dengue proteins. We used a two-hybrid matrix mating assay to test all 14 DBD fusion proteins against all 14 AD fusion proteins (Materials and Methods). We detected interactions between NS5 and both NS3 and NS3Δ1–160 (Figure S1). The interaction between NS5 and the C-terminal region of NS3, which contains the helicase domain, was previously demonstrated by yeast two-hybrid and co-immunoprecipitation assays [27], [55] and NS3 was shown to be associated with cytoplasmic NS5 in dengue-infected cells [56]. The complex of NS3 and NS5 may be essential for viral replication since NS3 contributes the helicase to unwind a viral dsRNA intermediate allowing NS5 to synthesize a new RNA molecule. We also detected an interaction between the NS5 DBD and NS5 AD clones suggesting a homodimer. An NS5 homodimer was also observed in another yeast two-hybrid study [57]. No other novel interactions were detected. We failed to detect previously reported interactions between NS2B and NS3 [58] or between PrM and E, which were originally detected by co-purification assays and not by yeast two-hybrid [27], [58], [59]. It was not possible to detect the interaction corresponding to that reported between the precursor proteins NS2B-NS3 and NS4B-NS5 [60] because we did not express these fusion proteins or co-express NS2B with NS3, or NS4B with NS5 in our screen.

### Dengue-mosquito protein-protein interactions

To identify mosquito proteins that interact with dengue proteins, we constructed a yeast two-hybrid AD library for *Aedes aegypti* using mRNA pooled from ten stages of development ranging from egg to adult (see Materials and Methods). We used a library mating assay to screen the mosquito library with each of 14 individual dengue bait proteins (Materials and Methods). To verify two-hybrid interactions, we subcloned the mosquito cDNAs from initial positives into new AD vectors and retested for interaction with the original dengue bait proteins. At the same time we tested for interactions with baits unrelated to the dengue baits to identify proteins that may nonspecifically interact with random proteins. In all we identified 102 interactions that were reproducible and specific by this definition (Table S1). These interactions involved eight viral bait proteins representing C, NS3, NS5, or variants of these proteins and PrM (Table 1). We did not find mosquito proteins interacting with the membrane proteins M, E, NS2A and NS2B, or with the luminal proteins Eiii and NS1. This is likely because these proteins are unable to locate or fold properly in the yeast nucleus, consistent with data from other large-scale two-hybrid screens that are generally depleted for membrane proteins [42], [61], [62]. None of the mosquito–dengue interactions that we identified had previously been identified.

**Table 1:** Number of host interactors for each dengue protein identified in this study by yeast two-hybrid screens.

Dengue Protein	Mosquito	Human
C	16	20
PrM/M	1	0
NS3	34	15
NS4A	1	0
NS4B	1	0
NS5	49	11
E, NS1, NS2A, NS2B	0	0

The 102 interactions involved 93 unique mosquito proteins, 58 of which have clear human orthologs. Two of the mosquito-dengue interactions that we detected had been previously detected for the human orthologs (Table S2). These included NS5 interactions with the mosquito E3 ubiquitin ligase Seven In Absentia (AAEL009614) and the human Seven In Absentia Homolog, SIAH2 (ENSG00000181788), which was previously detected by Le Breton et al. [34]; and the interactions between NS5 and mosquito Paramyosin (AAEL010975) and human cingulin like-1 (ENSG00000128849) previously detected by Khadka et al [33]. None of the other mosquito-dengue interactions that we detected have human-dengue counterparts found in other studies. While some of these may be genuine species-specific dengue interactions, it is also likely that the lack of overlap with previous studies is largely due to differences in the techniques and libraries used. We used library screening and directed assays (described further below), to detect 9 additional human-dengue interactions that correspond to 8 of the mosquito-dengue interactions, indicating that at least some of the mosquito-dengue interactions may be conserved (Table S1). It has been reported that some human proteins interact with proteins from a range of different viruses, perhaps because these human proteins are common viral targets or part of common cellular responses to viral infections [33], [63]. We found that 15 of the mosquito proteins that we identified have human orthologs that interact with other viral proteins (Table S2). These include several that could be considered conserved interactions or interologs because they involve orthologous proteins from Hepatitis C virus (HCV). For example, we detected an interaction between dengue NS3 and mosquito titin (AAEL002565), an ortholog of human obscurin (OBSCN), which was shown to interact with HCV NS3 in a large-scale two-hybrid screen [64]. Similarly, we detected an interaction between NS3 and mosquito nucleosome assembly protein (AAEL005567), an ortholog of human Nucleosome Assembly Protein 1-Like 1 (NAP1L1), which was shown to interact with HCV NS3 [64]. The NS3 proteins from both dengue and HCV contain serine protease and RNA helicase domains and function similarly during the maturation of the viruses [17].

We looked at the expression data available for the mosquito genes that we identified. Several studies have examined the transcription response of mosquito tissues or cells to infection with dengue [65]–[67]. We found that genes for relatively few of the dengue interacting proteins appeared to be regulated in response to dengue infection. Among the transcripts that were modestly downregulated in the mosquito carcass in response to dengue [66], were four encoding NS3 interactors: three heat shock proteins (AAEL014845, AAEL014843, AAEL011708) and an uncharacterized ATPase (AAEL010585) that is orthologous to human valosin containing protein (VCP or p97). Interestingly, VCP, which is involved in protein processing in the endoplasmic reticulum [68], [69], is also required for replication of poliovirus [70] and has been linked to the pathogenesis of hepatitis B virus [71]. None of the dengue interactors were shown to be regulated in midgut after infection [65]. Another study identified genes that are regulated in response to dengue infection in salivary glands, a key site of secondary infection in the mosquito [67]. Transcripts for two of the interactors are upregulated by dengue in the salivary gland, including one of the NS3-interacting heat shock proteins (AAEL014843) and a carboxypeptidase (AAEL010782) that interacted with capsid. Expression studies have found that genes associated with the innate immune response are regulated in response to dengue infection, including genes in the Toll-like receptor pathway and the JAK/STAT pathway [66]. We did not identify interactions involving any of these regulated genes. However, we did find that NS3 and NS5 interacted with endoplasmin (AAEL012827), a heat shock protein in the endoplasmic reticulum. Transcripts for endoplasmin are highly enriched in the salivary gland [67]. Endoplasmin, also known as gp96, is a chaperone required for proper folding of Toll-like receptors [72]–[74]. It is tempting to speculate that the interactions of endoplasmin with NS3 and NS5 may be a primary step in the innate immune response to dengue infection, or in the virus's attempt to circumvent the response.

In all we identified 34 NS3-interacting mosquito proteins. To explore the NS3 domains that may interact with the host proteins, we tested all of them against both full-length NS3 and NS3Δ1–160 (Table S3). As expected, all of the host proteins interacted with full-length NS3, including the three proteins that were originally isolated with NS3Δ1–160. Interestingly, most host proteins also interacted with NS3Δ1–160, indicating that they interact with the C-terminal half of NS3, which contains the helicase domain. Five host proteins were incapable of interacting with NS3Δ1–160, suggesting that they require the N-terminal protease domain of NS3 for interaction (Table S3). The NS3-interacting proteins were enriched for proteins with the gene ontology annotation “response to stress” and for proteins with the domain “heat shock protein” (Table S4), primarily because they include several heat shock proteins. Human Hsp90 and Hsp70 have been found in the dengue virus receptor complex [75], but no intracellular role for heat shock proteins during virus replication has been reported.

We identified 49 NS5-interacting mosquito proteins. The top enriched domains among these interactors were associated with myosin, found in non-muscle or smooth muscle myosin heavy chain (AAEL005656 and AAEL005733), myosin v (AAEL009357), long form paramyosin (AAEL010975), and a hypothetical protein (AAEL014104) (Table S4). Although there is no evidence linking myosin and NS5, myosin Vc was reported to be involved in the release of dengue virus from HepG2 cells [76]. Colpitts et al., detected several myosin proteins by co-affinity purification from mosquito cells; however, NS5 was not used in their study [36].

We identified 16 capsid-interacting mosquito proteins. Three of these were identified using the anchored capsid bait that contained the C-terminal membrane-spanning domain, while the others were identified using the mature capsid bait. We tested all of the capsid-interacting proteins to see if they were capable of interacting with each capsid bait protein and found that all but two proteins were capable of interacting with both baits (Table S5). The two-hybrid reporter activity was generally less with the anchored capsid compared to the mature capsid, which could explain our failure to isolate these proteins with the anchored capsid bait even though they were capable of interacting with it. This could be due to a lower expression level of the anchored capsid or to the impaired ability of the membrane domain to enter the yeast nucleus and fold properly. The capsid-interacting mosquito proteins are enriched for “nucleic acid binding” proteins and proteins with “Zn finger” domains (Table S4). Among the nucleic acid binding capsid-interacting proteins we three potentially RNA-binding proteins according to the functions of their human orthologs: a hypothetical protein (AAEL011985), putative myosin I (AAEL003676), and DEAD box ATP-dependent RNA helicase (AAEL009285). Moreover, the top protein domain enriched among the capsid interactors was the “G-patch” domain, which functions as an RNA-binding domain found in mRNA processing proteins and some retroviruses [77], [78]. Since dengue capsid also directly binds to viral genomic RNA [79], it may be interesting to investigate whether interaction between capsid and G-patch proteins has any role in packaging the genome into the viral particle.

### Dengue-human protein-protein interactions

While dengue-human protein interactions have been explored more extensively than dengue-mosquito protein interactions, the lack of overlap among validated interactions from different screens suggests that the dengue-human interactome is still incomplete. To complement the dengue-mosquito interactome and other dengue-human studies, we conducted two-hybrid screens using the 14 dengue protein baits (Figure 1B) and a cDNA library from human peripheral blood leukocytes (PBL). PBL contains a population of cells of the mononuclear phagocyte lineage, which are the primary target of dengue virus infection in human [80]–[82]. The library screens and the reproducibility and specificity tests were conducted as in the mosquito library screens (Materials and Methods). Similar to the mosquito library screen, we did not find human proteins interacting with M, E, NS1, NS2A, NS4A and NS4B; nor did we find interactors for PrM. In total we identified 46 reproducible specific interactions between 35 human proteins and five bait proteins representing variants of dengue C, NS3, and NS5 (Table 1 and Table S6). Only six of the interactions had previously been detected or predicted (Table S6). These included two interactions (Capsid – Beta Hemoglobin (HBB) and Capsid - Ribosomal Protein L5 (RPL5)) that had been predicted based on structural similarity between dengue virus and host proteins [37] and four interactions (NS3 – Nuclear Factor of Kappa light polypeptide gene enhancer in B-cells Inhibitor, Alpha (NFKBIA), NS3 – Nuclear Receptor Binding Protein 1 (NRBP1), NS3 – Golgin B1 (GOLGB1), and NS5 – Rab Interacting Lysosomal Protein-Like 2 (RILPL2)) that were identified in separate two-hybrid screens [33], [34], [83].

Our library screens identified four putative conserved interactions, where both human and mosquito orthologs were identified as interacting with the same dengue proteins (Table S1). 54 out of the remaining 93 mosquito genes that we identified have human orthologs that we failed to isolate in screens of the human PBL library. Our failure to isolate human orthologs of these 54 mosquito genes could be because they are missing from the PBL cDNA library, or because the human orthologs actually do not interact with dengue proteins. To distinguish between these possibilities and to identify additional human-dengue interactions, we set out to test whether human orthologs of the mosquito proteins interact with the same dengue protein. Sequence analysis identified 96 potential human orthologs for the 54 mosquito genes (Table S1). We were able to retrieve and subclone 55 of these from a library of full-length human ORFs [84]. These 55 human genes are potential orthologs of 31 mosquito genes. We made two-hybrid AD clones for these 55 full-length ORFs and screened them against the corresponding dengue virus proteins. This resulted in identification of an additional five human-dengue interactions corresponding to four of the mosquito-dengue protein interactions (Table S1).

Combined, our human cDNA library screens and directed assays of mosquito orthologs identified 52 interactions involving 47 human proteins and three dengue proteins, capsid, NS3 and NS5 (TableS1 and Table S6). These include 46 novel interactions that were not previously detected or predicted; nine of these were detected with both human and mosquito orthologs. A global analysis of the human dengue-interacting proteins reveals no enriched GO annotations or protein domains. Similar to our finding with the mosquito proteins, a significant proportion of the dengue-interacting human proteins (19 out of 47) have been shown to interact with proteins from other viruses (Table S7) [85]–[91]. These include at least two interactions that could be thought of as conserved: Dengue NS3 interacted with Zinc Finger Protein 410 (ZNF410) and Calcium Binding and Coiled-Coil domain 2 (CALCOCO2), both of which have been shown to interact with HCV NS3 by a two-hybrid screen [64].

It has been shown that proteins from other viruses frequently interact with hub proteins, which are host proteins that have a large number of interactions in the host interactome [63]. To evaluate the numbers of interactions for the host proteins that we identified, we assembled a human protein interactome from several public databases (Materials and Methods). The human interactome contains 43 (92%) of the 47 dengue-interacting proteins that we identified. It also contains 144 (89%) of the 161 dengue-interacting human proteins identified exclusively in other screens [22], [24], [27]–[30], [32]–[35], [55], [75], [92]–[105], and 53 (84%) of the 63 human orthologs of mosquito proteins that we identified. For each of these gene sets, we found that the average number of interactions (or degree) per protein was significantly higher than for random samples of similar numbers of proteins. For example, the average degree of dengue-interacting proteins in our dataset was 44.0, whereas the average degree of similarly sized random samples of proteins was 22.4 (*p*-value = 9.3×10^−4^) (Figure S2). The dengue interactors from mosquito were also enriched for proteins with many interactions (*p*-value = 2.7×10^−3^), as were the dengue-interacting proteins identified by other studies (*p*-value = 6.4×10^−7^) (Figure S2). It has been suggested that the tendency of viral proteins to interact with hub proteins may represent a feature of viral pathogenesis, since the disruption of a hub is more likely to impair the cell's protein network than the disruption of a non-hub [63], [106]. While our results are consistent with this hypothesis, they could also be explained by the possibility that some proteins are particularly interactive in the protein interaction assays that have been used to detect the human interactome, including yeast two-hybrid. Thus a more thorough test of the hypothesis that dengue viral proteins tend to target hubs will require a larger set of functionally validated dengue-host interactions.

We identified a number of potentially relevant human NS3 interactors. CALCOCO2 (also known as NDP2) is a component of Nuclear Domain 10 (ND10) bodies, which play a role in the intrinsic cellular defense mechanisms against some viruses [107]. Interestingly, another major component of ND10 is DAXX, which has been shown to interact with dengue capsid [30]. Another NS3 interactor, Osteosarcoma amplified 9 (OS9), plays an important role in the unfolded protein response (UPR) [108], which is often observed in dengue-infected cells [109]. NS3 also interacted with three proteins that play roles in the Toll-like receptor-signaling pathway, which is important for the innate immune response to infection with many viruses [110], [111]. This is the case also in mosquito where the homologous Toll pathway is known to be important for the immune response to dengue. It is has been shown, for example, that a negative regulator of the Toll pathway, Cactus, (AAEL000709), is downregulated in response to dengue infection, while RNAi silencing of Cactus can reduce dengue infection [66]. It is still unclear, however, how or whether dengue proteins directly interface with the Toll pathway in mosquito or with the Toll-like receptor pathway in humans. The interactions that we identified with NS3 may provide clues. One NS3 interactor that we found, as did Le Briton et al. [34], is a human ortholog of Cactus, NFKBIA, a protein that inhibits Toll-like receptor signaling by inhibiting the NF-kappaB transcription factor [112], [113]. Another NS3 interactor that we identified is Coronin-1 (CORO1A), an actin-binding protein that is capable of inhibiting Toll-like receptor signaling [114]. Finally, we found that NS3 interacted with a protein named Leucine Rich Repeat (in FLII) Interacting Protein 1 (LRRFIP1). LRRFIP1 is a regulator of the Toll-like receptor-signaling pathway and has been shown to associate with dsRNA-containing endosomes/lysosomes [115], which are generated in response to viral dsRNA intermediates during replication [116]. It seems likely that at least some of these human NS3-interacting proteins may be involved in the interplay between the host defense mechanisms and the viral strategies to circumvent them. As we did for the mosquito proteins, we tested the human NS3 interacting proteins against both variants of NS3 baits (Figure 1B) and found that the N-terminal 160 amino acids of NS3 was required for only three interactions, including the previously identified interaction NS3 – NRBP1 (Table S3). The remaining human proteins interacted with both the full-length and the C-terminal half of NS3.

The capsid interactors were isolated using the anchored capsid bait or the cytoplasmic capsid bait (Figure 1B). We tested all of the capsid interactors against both baits and found that most were able to interact with both the anchored and the cytoplasmic capsid proteins (Table S5), indicating that the C-terminal membrane spanning domain is not required for and does not dramatically interfere with most interactions. GO and protein domain enrichment analysis of the 20 capsid interactors failed to implicate any specific biological function or process (Table S8). However, the capsid interactors include a preponderance of ribosomal proteins, including RPL5, RPL6, RPL7, and RPL27, all of which are subunits of the 60S ribosome. Capsid also interacted with mosquito RPL23, and with a ribosomal RNA processing protein (RRP12) from both mosquito and human. Based in part on these ribosomal proteins and on a GTP-binding protein, GTPBP4, the capsid-interacting proteins are enriched for proteins annotated as being associated with the nucleolus (Table S8). Interestingly, dengue capsid has previously been found to accumulate in nucleoli in several cell lines [117]–[119], though the functional significance of this localization has not been determined. Many other viruses interact with nucleoli, and in some cases nucleoli have been shown to be essential for virus replication [120], [121]. The capsid proteins from two other flaviviruses, West Nile virus and Japanese encephalitis virus, for example, each interact with specific nucleolar proteins, and in each case, these nucleolar proteins have been shown to be important for efficient viral replication [122]–[124]. Further studies with the proteins that we identified to interact with dengue capsid may provide insights into the role and mechanisms for accumulation of dengue capsid in the nucleolus.

Among the human NS5 interactors, “unfolded protein response (UPR)” is the top enriched GO annotation (Table S8). This enrichment is based on interactions with DERL2, an ER membrane protein involved in targeting misfolded glycoproteins for degradation [125], [126], and HSPA5/Grp78/BiP, an ER protein involved in protein folding [127], [128]. The UPR is known to be activated during dengue infection; however, its importance for virus replication is still undetermined [109], [129]–[131].

### Confirmation of protein interactions using additional assays

**Figure 2:**
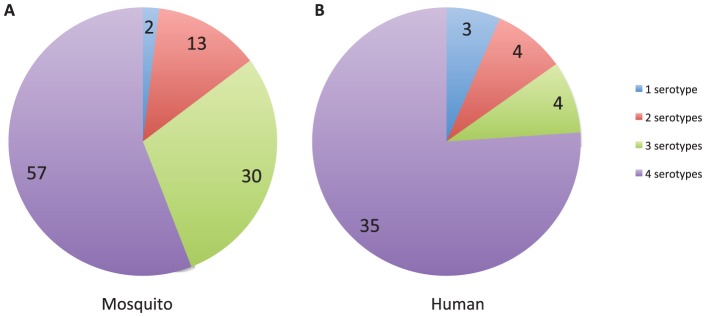
Pie charts showing the number of host proteins that interacted with the corresponding proteins from one, two, three, or all four dengue serotypes. (A) Mosquito proteins. (B) Human proteins.

Yeast two-hybrid studies frequently detect false positive interactions that have no biological relevance. One way to gain confidence in a two-hybrid interaction is to detect it using additional assays. We used two approaches to test the confidence of interactions that we identified in the library screens. First, we reasoned that biologically relevant virus-host protein interactions are likely to be conserved across the four dengue serotypes. There is 63–68% amino acid sequence homology among the four serotypes [17]. An interaction between a host protein and the same dengue protein from multiple serotypes may imply that the interaction is more likely to have functional relevance because significant variation in the dengue protein does not interrupt the interaction. To test for conservation of interactions we repeated two-hybrid assays for all dengue-host interactions using dengue proteins from serotypes 1, 3 and 4. We found that 57 out of 102 (56.9%) dengue-mosquito protein interactions and 34 out of 46 (73.9%) dengue-human protein interactions were serotype independent; i.e., the host proteins interacted with the corresponding dengue proteins from all four serotypes (Figure 2; Tables S1 and S6). This provides additional evidence that these host proteins genuinely interact with the dengue proteins, and further points to conserved sequences or structural elements in the dengue proteins as potential interaction interfaces. A minority of the host proteins interacted with only one or a subset of the dengue serotypes (Figure 2; Tables S1 and S6). While some of these interactions may be false positives, others may be biologically relevant serotype-specific dengue-host interactions. If so, such interactions may mediate some of the serotype-specific dengue characteristics that are clinically observed [132]. Further investigation will be required to validate serotype specific interactions.

**Figure 3:**
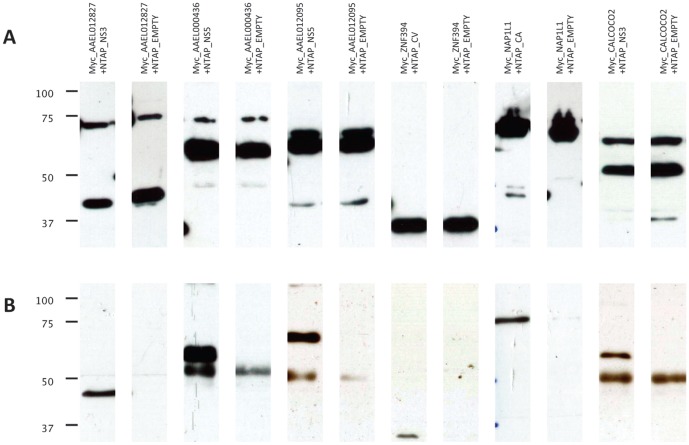
Examples of co-AP results. Host proteins were fused to a myc-tag while dengue proteins were fused to an NTAP-tag. The fusion proteins were expressed in S2R+ cells. NTAP-dengue proteins were purified from cell lysates, and then host proteins were detected with α-myc. (A) An α-myc immunoblot of cell lysates shows expression of mosquito and human proteins. (B) An α-myc immunoblot of NTAP-tag affinity-purified samples. Additional co-AP results are in Figure S3.

Next we employed an orthogonal assay, co-affinity purification (co-AP), to test most of the dengue-host interactions that we identified by yeast two-hybrid assays. We expressed myc-tagged versions of the mosquito and human proteins in cultured *Drosophila* cells along with NTAP-tagged dengue proteins (Materials and Methods). We purified the tagged dengue proteins and tested for co-purification of the host proteins by immunoblotting with myc antibodies (Figure 3 and Figure S3). If one of the two proteins failed to express in the cell lysate, we tried the experiment in the opposite orientation by expressing the dengue protein with a myc tag and the host protein with an NTAP tag. We were able to express and test by co-AP 136 pairs of proteins, and we detected 38 interactions (27.9%) (Table S1 and S6). This confirmation rate is similar to that reported for other large-scale tests of protein interactions by orthogonal assays [133], [134], but lower than the rate reported in some individual two-hybrid studies [61], [135]. One possible explanation for the discrepancy is that we define a two-hybrid positive based on reproducible activity of a highly sensitive *LEU2* reporter, and thus we may detect weaker protein-protein interactions than studies that require activation of multiple less sensitive two-hybrid reporters. The combined two-hybrid reporter activity (*LEU2* and *lacZ*) that we observed was only slightly higher for interactions that were positive by co-AP assays (average 3.4) than for interactions that were negative in co-AP assays (average 2.9), but this difference was not statistically significant. Thus, the numbers of active reporters and their levels in the two-hybrid assay was not a strong predictor of reproducibility by co-AP assay.

**Figure 4:**
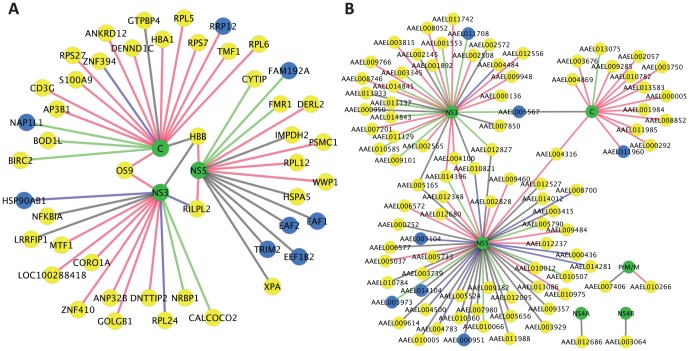
Dengue – host protein networks derived from two-hybrid screens and co-AP assays in this study. (A) Human-dengue interaction map. (B) Mosquito-dengue interaction map. Edges represent protein-protein interactions. Green nodes are dengue proteins, yellow nodes are host proteins, and blue nodes are host proteins found in both the human and mosquito maps. Red edges represent protein-protein interactions universally detected for all four serotypes. Blue edges represent protein-protein interactions confirmed by co-AP assays. Green edges represent the universal interactions that were confirmed by co-AP assays. Additional details are available in Cytoscape files in supplemental data (Data S1).

Figure 4 shows a summary of the dengue-host interactions that we identified. The dengue-human interaction map includes 13 proteins that have orthologs in the dengue-mosquito map and that were involved in protein-protein interactions (PPI) in both species. Three human proteins and seven mosquito proteins interacted with more than one dengue protein. The maps also show whether or not each interaction was detected with all four dengue serotypes and whether or not it was also detected by co-AP.

### A snapshot of the dengue-host interactome

It is often noted that a virus such as dengue with only 10 proteins of its own should need to interact with a number of host proteins to carry out its replication cycle. Our study combined with other large-scale and small-scale studies [22], [24], [27]–[30], [32]–[36], [55], [75], [83], [92]–[105] have identified 403 interactions between proteins from dengue and its hosts, not counting the more than 4,000 interactions that have been computationally predicted [37]. Since we know that most protein interaction screens and assays produce false positives, it seems likely that a number of the dengue-host PPI detected thus far are not relevant to the virus or the host's defenses against it. Among the 403 experimentally detected PPI, only seventeen PPI [23], [28], [33], [93], [94], [100], [103]–[105] have been studied further and shown to potentially have functional significance. How then can we decide which of the remainder of the interactions merit further investigation? The number of validated PPI is too small to use as a gold standard for developing a statistical scoring system to rank all PPI, as has been done for other interactomes [48], [50], [136]. Thus, we propose the use of two criteria for prioritizing the dengue-host PPI for further study. The first criterion is based on the observation that PPI detected by multiple independent assays or studies are more likely to be biologically relevant [42]–[47], [137]. Assuming that this is also true for the dengue-host interactions, we counted the number of assays and the number of studies that detected each of the physical interactions. Any orthogonal assay was counted as an individual piece of evidence. Two similar assays detecting the same PPI, but conducted by two independent groups, was also counted as two pieces of evidence. By this criterion, 67 of the 403 dengue PPI were detected thus far by more than one assay or study.

**Figure 5:**
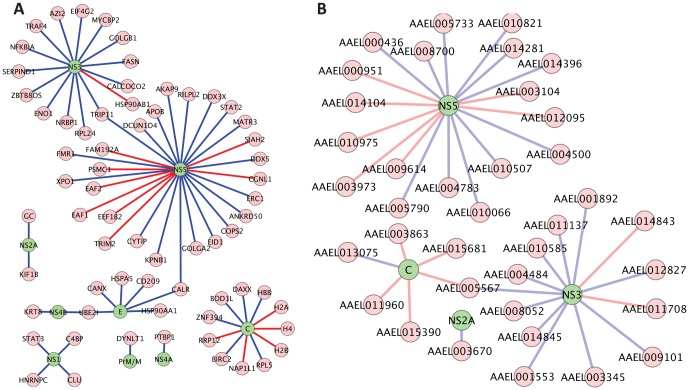
Dengue-host interactions supported by multiple forms of evidence. (A) Dengue-human interactome. (B) Dengue-mosquito interactome. Pink nodes represent host proteins. Green nodes represent dengue proteins. Red edges represent PPI with conserved interologs. Additional details are available in Cytoscape files in supplemental data (Data S1).

The second criterion proposed here is based on the fact that many biologically relevant PPI are conserved [138], and thus detection of the same interaction in two different species is tantamount to detecting the interaction more than once. Applying this criterion to the dengue-host interactions, we counted a PPI as a potentially conserved interolog if it was found in both mosquito and human. 28 PPI (14 PPI for each species) were detected in both species. We also counted an interaction as having multiple forms of supporting evidence if it was experimentally detected and also computationally predicted [37]. Taking these criteria together, we derive a list of 35 dengue-mosquito PPI and 65 dengue-human PPI that have multiple forms of supporting evidence (Figure 5; Table S10). The list is biased against dengue and host membrane proteins, largely because the primary techniques that have been used to detect protein interactions, yeast two-hybrid and complex pull downs, are poor at detecting interactions involving membrane proteins [62]. The application of additional methods, such as protein fragment complementation [139] or co-complex purifications optimized for membrane proteins [140], will likely enable expansion of the list both by identifying new dengue-host interactions and by providing additional evidence for interactions that were thus far detected by only one method. In the meantime, using multiple forms of evidence as a guide can prioritize the existing protein interactions that merit further investigation.

For the above analysis we considered only evidence of direct protein-protein interactions. An alternative approach would be to consider other forms of evidence that may support the involvement of specific host proteins in dengue infection. Strong supporting evidence for a functionally relevant protein interaction, for example, can come from experiments showing that the host protein is required for efficient infection or propagation of the virus. Below we consider a number of protein interactions that, although detected by only one method, are supported by such functional data. Khadka et al. [33] used RNAi to test whether the expression of several human dengue-interacting proteins was required for efficient replication of a transfected dengue replicon. They showed that knockdown of six human proteins (CALR, DDX3X, ERC1, GOLGA2, TRIP11, and UBE2I) reduced the expression of a dengue reporter gene. Interactions involving two of these proteins (UBE2I and CALR) are supported by multiple forms of evidence (Figure 5A), yet the functional assay added additional support, particularly for interactions that had only been detected by the yeast two-hybrid assay. These include GOLGA2 – NS5, ERC1 – NS3, TRIP11 – NS3, TRIP11 – NS5, and the interaction of DDX3X with E, NS2A, NS4B, and NS5. Support for the functional significance of protein interactions has also come from genome-wide RNAi knockdown studies. Krishnan et al., [141] identified genes that when knocked down enhanced or inhibited infection of human cells with West Nile Virus, and then retested the hits for their effects on DENV2 infectivity. That study lends additional support to two interactions from our study (DERL2 – NS5 and ANP32B – NS3), and to another interaction (VPS11 – NS5), originally detected by two-hybrid [34]. In another study, Sessions et al., [142] identified *Drosophila* genes that were required for DENV2 propagation in *Drosophila* cells and then tested human orthologs of a subset of the hits in a similar assay. That study provided functional evidence supporting several protein interactions that had been detected only by yeast two-hybrid assays, including ENOX2 – NS3 [34], and as noted by Khadka et al. [33], SCLT1 – NS2A, ERC1 – NS5, and TTC1 – NS5. The genome-wide knockdown studies also support two interactions that we detected with mosquito genes, AAEL003750 – C and AAEL004783 – NS5. Knockdown of the human orthologs of AAEL003750, nucleoplasmin 3 (NPM3), enhanced dengue infection in human cells [141]; Guo et al., previously used this data to predict the involvement of AAEL003750 in dengue infection [38]. Knockdown of the *Drosophila* ortholog of AAEL004783, ornithine decarboxylase antizyme (Oda), inhibited propagation of dengue in *Drosophila* cells [142]. These examples show how integration of physical protein interaction data with functional data from high throughput studies can be used to prioritize host-dengue protein interactions for further analysis. Further investigation of these high-priority PPI and those supported by multiple forms of evidence (Figure 5) may lead to a better understanding the interplay between dengue and its hosts. Furthermore, these data should be useful for identifying host proteins or pathways that may be candidates for drug targets and vector control strategies.

## Materials and Methods

### Plasmids and subcloning

pHZ12 and pHZ13 are vectors containing the Gal4-responsive upstream activating sequence (UAS) driving expression of proteins with an N-terminal myc tag or TAP tag. Both vectors were made by modifying pUASattB [143], which contains the *Drosophila* marker gene mini-*white* and the phiC31 phage attachment site (attB) enabling targeted integration into specific attP sites engineered into *Drosophila* chromosomes. To construct pHZ12, a PCR fragment from pAS1 [144] was inserted downstream of the UAS in pUASattB. The PCR fragment contained a *Drosophila* translational start sequence followed by codons for 6 histidines and 3 myc tags, and recombination target sequences, 5RT (5′-TTGACTGTATCGCCG-3′) and 3RT (5′-CCGGAATTAGCTTGGCTGCAG-3′); these sequences allow insertion of open reading frames (ORFs) using recombination in *E. coli* or yeast [145]. pHZ13 was constructed by inserting a fragment containing the NTAP tag from pUAS-NTAP [146] followed by 5RT and 3RT downstream of the UAS in pUASattB. pHZ12_attR and pHZ13_attR were constructed by inserting a Gateway destination vector cassette (Invitrogen) into the cloning sites of pHZ12 and pHZ13. Briefly, the Gateway cassette was PCR amplified from pJZ4_attR with primers, DM138 and DM139 (see Table S9 for sequences) and then digested with *Xba*I and inserted into pHZ12 and pHZ13 digested with *Pme*I and *Xba*I. The ligations were used to transform *E. coli*, OmniMAXII (Invitrogen). Transformants with plasmids containing a Gateway cassette were selected on LB-*Chloramphenicol/Ampicillin* media. pJZ4_attR is a gateway destination plasmid containing an activation domain for yeast two-hybrid assays [147].

Dengue virus prototype strains for serotypes 1 (Hawaii), 2 (16681), 3(H87) and 4 (H241) were cultured in C6/36 cell lines. Viral RNA was extracted from the culture supernatant with the QIAamp Viral RNA Kit (Qiagen). cDNAs encoding each dengue virus 2 protein were generated from an infectious clone, a gift from Dr. Nopporn Sittisombut (Chiang Mai University, Chiang Mai, Thailand). cDNA synthesis was performed with primers targeting the 3′ end of each viral genome – Hawii and 16681 CACCATTCCATTTTCTGGCGTTC, H87 TGGCGTTCTGTGCCTGGAATGAT and H241 TCAACAACACCAATCCATCTTGCGG - using SuperScript II Reverse Transcriptase (Invitrogen) according to the product manual. Individual viral genes or gene fragments were PCR amplified from the cDNA with specific primers (Table S9). Each 5′ primer included the attB1 sequence, 5′ – GGGG-ACA-AGT-TTG-TAC-AAA-AAA-GCA-GGC-T – 3′, and each 3′ primer included the attB2 sequence, ′5 – GGGG-ACC-ACT-TTG-TAC-AAG-AAA-GCT-GGG-T– 3′. The PCR products were cloned into pDONR221 and then into destination vectors using BP Clonase II and LR Clonase (Invitrogen) according to the manufacturer's protocol. The entry clones were sequenced with M13F(-21) and M13R sequencing primers. Each viral gene or gene fragment was transferred to the following destination vectors: pNLex_attR and pJZ4_attR for two-hybrid assays [147], and pHZ12_attR and pHZ13_attR for co-AP assays. Human open reading frames (ORFs) were similarly cloned into these destination vectors from entry vectors obtained from the human ORFeome collection [84] obtained from Open Biosystems. For co-AP assays, individual mosquito or human cDNAs were subcloned from the yeast two-hybrid library vectors into pHZ12 and pHZ13 by recombination cloning as previously described [145].

### Mosquito cDNA libraries for yeast two-hybrid screens

An *Aedes aegypti* colony was established and maintained according to a published protocol [148]. Briefly, a mosquito colony was maintained at 27°C, 70–90% relative humidity in an 8-hour dark/16-hour light cycle. Newly hatched larvae were fed with ground rat food. One-day-old larvae were counted, and a group of 200–250 larvae were added in a tray containing 700–800 ml of water. Three pellets of cat food (Friskies Senior) were added to each tray. Adults were fed with either 10% sucrose or blood from a mouse. RNA was isolated separately from ten stages of development including 1) less than three-month-old embryos, 2) one-day-old larvae, 3) two-day-old larvae, 4) three-day-old larvae, 5) four-day-old larvae, 6) five-day-old larvae, 7) six-day-old larvae, 8) pupae, 9) adults and 10) adults collected three hours after a blood meal. In order to collect enough RNA, eggs from several layings were independently collected and pooled; the oldest eggs in the pool were aged less than three months. Eggs were synchronously hatched by applying a vacuum (13 to 15 inHg) for 20–40 minutes. Larvae were collected every 24 hours for six days. Pupae were collected at 120–144 hours after egg hatching. Adults were collected 3 days after emerging from pupae. The adults fed with a blood meal were collected three hours later so that the genes responding to blood ingestion were sufficiently expressed [149]. Samples were homogenized with a dounce homogenizer and RNA was isolated from tissue homogenate with an RNeasy Midi kit (Qiagen). The RNA was treated with RNase-free DNase (Qiagen) at room temperature for 15 minutes. Poly(A) RNA was enriched with the Poly(A)Purist kit (Ambion). 0.5 µg of poly(A) RNA from each of the ten stages was pooled and used for cDNA synthesis with the directional cDNA Synthesis kit (Stratagene), which uses an oligo dT 3′ primer containing an *Xho*I site, 5′- GAGAGAGAGAGAGAGAGAGAACTAGTCTCGAGTTTTTTTTTTTTTTTTTT -3′. Next, the cDNA was ligated with an *EcoRI* adapter, 5′-OH-AATTCGGCACGAGG-3′ 3′-GCCGTGCTCCp-5′

The cDNA was digested with *Xho*I, fractionated on Sepharose CL-2B gel filtration medium and then ligated into the yeast two-hybrid activation domain (AD) vector, pRF4-5o [150] cut with EcoRI and XhoI. Ligation reactions were ethanol precipitated, washed in 80% ethanol, resuspended in sterile distilled water, and then used to transform *E.coli*, *MegaX DH10B*™ T1R Electrocomp™ Cells (Invitrogen), by electroporation. 188 *E. coli* transformant colonies were randomly picked for colony PCR. 64% of the colonies had inserts with various sizes from 300 to 4,000 bp. The average size of the inserts was 1,400 bp. More than 10^7^ *E. coli* colonies were collected for plasmid extraction with a QIAGEN Plasmid Giga kit.

### Yeast two-hybrid screens and assays

The LexA version of the two-hybrid system from the Brent laboratory [151] was used to screen for host proteins (human or mosquito) that interact with LexA-fused dengue proteins essentially as described [152] (Table S10). A summary of the screening results is available in Table S11. The mosquito cDNA library (described above) was used to transform yeast strain RFY231 (MATα trp1::hisG his3 ura3–1 leu2::3Lexop-LEU2) using the lithium acetate method [153] and Trp+ colonies were selected. 1.1×10^8^ transformant colonies were harvested and frozen in 1 ml aliquots at −80°C. Similarly, a human cDNA library (Origene Technologies) with cDNA from peripheral blood leukocytes (PBL) cloned into the AD vector pJG4–5 [154] was used to transform RFY231 and 2.2×10^8^ transformant colonies were harvested and frozen in aliquots. Bait strains were created by transforming RFY206 (MATa trp1Δ::hisG his3Δ200 leu2–3 lys2Δ201 ura3-52 mal-), which also contained the *lacZ* reporter plasmid, pSH18-34(URA3+), with pNLex bait plasmids expressing dengue proteins. Each screen was carried out by mating a freshly grown aliquot of a bait strain with a thawed aliquot of yeast containing the human or mosquito libraries. About 2×10^8^ colony-forming units (cfu) of the bait strain were mated with 0.5–1×10^8^ cfu of the library strains on a YPD plate. Diploids were collected, induced in liquid galactose medium for 4 hours at 30°C, and plated on galactose medium lacking leucine. cDNA inserts from galactose-dependent Leu+ yeast were PCR amplified and re-cloned into the AD vector in fresh RFY231 yeast by gap repair [155]. The freshly transformed yeast were then used in 96-well interaction mating assays as previously described [150], [156] to confirm an interaction with the original bait protein. To test for specificity, interactions were also tested between each host protein and two unrelated bait proteins, *Drosophila* Eip63E (FBgn0005640) and Cyclin J (FBgn0010317). cDNAs that passed the tests were PCR amplified and digested with *AluI* to identify unique digestion patterns, which may represent unique genes [152]. These cDNAs were then sequenced and identified. Human or mosquito proteins that were confirmed to interact with the dengue serotype 2 bait used to isolate them were also tested against the same dengue protein in all four serotypes using the 96-well interaction mating assays. cDNA from confirmed clones was PCR amplified, sequenced, and the genes were identified by nucleotide BLAST analysis.

### Co-affinity purification (Co-AP) assays

*D. melanogaster* S2R+ cells were obtained from the *Drosophila* Genomics Resource Center at Indiana University. The cells were cultured in Schneider's media (GIBCO) supplemented with 10% FBS (Gemini) and 1%Gentamycin (GIBCO). S2R+ cells were incubated at 25°C. The cells were passaged once per week at 1∶10 dilution. Co-AP assays were performed essentially as described [144]. Briefly, S2R+ cells were co-transfected with pHZ12 plasmids containing human or *Aedes aegypti* cDNA, pHZ13 plasmids containing dengue ORFs, and the expression driver plasmid pMT-GAL4 using the tranfection reagent, Effectene (Qiagen). pMT-Gal4 expresses Gal4 from the Cu^+2^-inducible metallothionine promoter [157]. 1×10^6^ cells were seeded onto each well of a 12-well cell culture plate 18–24 hours before transfection with 250 ng of each plasmid, pHZ12, pHZ13 and pMT-GAL4. 12–18 hours after transfection the media was supplemented with 1 mM CuSO_4_ to induce protein expression. Three days after CuSO_4_ induction the cells were harvested and lysed. Expression of fusion proteins was determined by Western blot analysis of cell lysates using anti-NTAP (Rockland Immunochemicals) and anti-Myc (Santa Cruz Biotechnology) antibodies for proteins expressed from pHZ13 and pHZ12, respectively. For lysates with detectable expression of both the Myc-tagged and NTAP-tagged proteins, IgG beads were used to purify the NTAP-tagged protein complexes, the complexes were run on SDS-PAGE gels, and the gels were immunoblotted with anti-Myc to probe whether the Myc-tagged protein was co-purified. For interactions involving proteins with undetectable expression, the co-AP assay was attempted by swapping the inserts between pHZ12 and pHZ13. Interactions were only considered tested if both tagged proteins could be detected in cell lysates.

### Computational analyses

For enrichment analysis of *Aedes aegypti* mosquito dengue interactors, a gene ontology annotation (GOA) file was downloaded from UniProt-GOA (ftp.ebi.ac.uk/pub/databases/GO/goa/proteomes/31436.A_aegypti.goa) [158], and an OBO file version 1.2 was downloaded from The Gene Ontology project (www.geneontology.org/ontology/obo_format_1_2/gene_ontology_ext.obo) [159]. A tree for InterPro domains was downloaded from EMBL-EBI (ftp.ebi.ac.uk/pub/databases/interpro/interpro.xml.gz) [160]. Cytoscape [161] with the BINGO plug-in [162] was used to analyze GO annotation and InterPro domain enrichments of dengue interactors. For analysis of the degree of dengue-interacting host proteins we assemble a human PPI interactome by combining the unique PPI collected from IntAct [163], MINT [164], BioGRID [165], Reactome [166] and HPRD [167] on 11 November 2010. The identifiers of the interacting proteins from these databases were all converted to Ensembl gene identifiers and a human PPI network having 134,050 unique interactions among proteins from 11,982 genes. Three gene sets were considered: human interactors identified in our screen, human interactors identified in other screens, and human orthologs of *Aedes aegypti* interactors identified in our screen. For each gene set the degrees of all the genes were calculated and averaged. Next, random sets of genes of equal size to each of the three gene sets were picked and the average degrees for each random set were calculated. This was repeated for one million random sets. The distribution of these averages is plotted in Figure S2. The *p*-value significance of the average degree for each gene set was calculated from the respective distribution using a two-tailed test.

## Supporting Information

Table S1 **Dengue-mosquito interactions found in this study.**
(XLSX)

Table S2 **Mosquito proteins with human orthologs that interact with proteins from other viruses.**
(XLSX)

Table S3 **NS3 domain analysis.**
(XLSX)

Table S4 **Gene Ontology (GO) and domain enrichment for mosquito proteins.**
(XLSX)

Table S5 **Capsid domain analysis.**
(XLSX)

Table S6 **Dengue-human interactions found in this study.**
(XLSX)

Table S7 **Human proteins that interact with proteins from other viruses.**
(XLSX)

Table S8 **Gene Ontology (GO) and domain enrichment for human proteins.**
(XLSX)

Table S9 **Oligonucleotide primers used in this study.**
(XLSX)

Table S10 **Dengue-host protein-protein interactions supported by multiple forms of evidence.**
(XLSX)

Table S11 **Yeast two-hybrid screen details.**
(XLSX)

Figure S1 **Intraviral protein-protein interactions.** Interactions were identified by the galactose-dependent growth of diploid yeast expressing two dengue proteins. Each panel is a group of four indicator plates: Glucose complete minimal (CM) lacking leucine (–leucine) (top-left), Galactose CM –leucine (top-right), Glucose CM +X-gal (bottom-left) and Galactose CM +X-gal (bottom-left). An interaction is indicated by galactose-dependent growth on the plates lacking leucine (top two plates in each panel) or galactose-dependent blue colony color on the X-Gal plates (bottom two plates in each panel). *Drosophila melanogaster* Cyclin Y and Eip63E were used as a positive interaction control while *D. melanogaster* Cyclin Y and Cyclin J were used as a negative control. All media lack uracil, histidine, and tryptophan to select the two-hybrid plasmids.
(PDF)

Figure S2 **Dengue-interacting host proteins are enriched for hubs.** Enrichment analyses for proteins with many interacting partners in three dengue-host interactomes: human interactors of dengue baits identified in this screen (A), human interactors of dengue baits identified in other screens (B), and *Aedes aegypti* interactors of dengue baits in this screen (C).
(PDF)

Figure S3 **Co-AP assays for dengue-host protein interactions.** Additional co-AP results that were not shown in Figure 3. Details are as described in Figure 3.
(PDF)

Data S1 **Cytoscape files containing all of the interaction data.** Four Cytoscape files combined into a single compressed file include all of the two-hybrid interactions detected between dengue and either human or mosquito proteins, as well as the interactions supported by multiple forms of evidence. The interactions are annotated with information about how they were detected, whether they were conserved among the four serotypes, and whether they were confirmed by co-affinity purification assays.
(ZIP)
