# Supplementary figures and images for: Identification of New Protein Interactions between Dengue Fever Virus and Its Hosts, Human and Mosquito
Source: PLoS One. 2013 Jan 11;8(1):e53535. doi: 10.1371/journal.pone.0053535 (PMC3543448; doi:10.1371/journal.pone.0053535)

Fig. S1

Mairiang et al. Supporting Figures

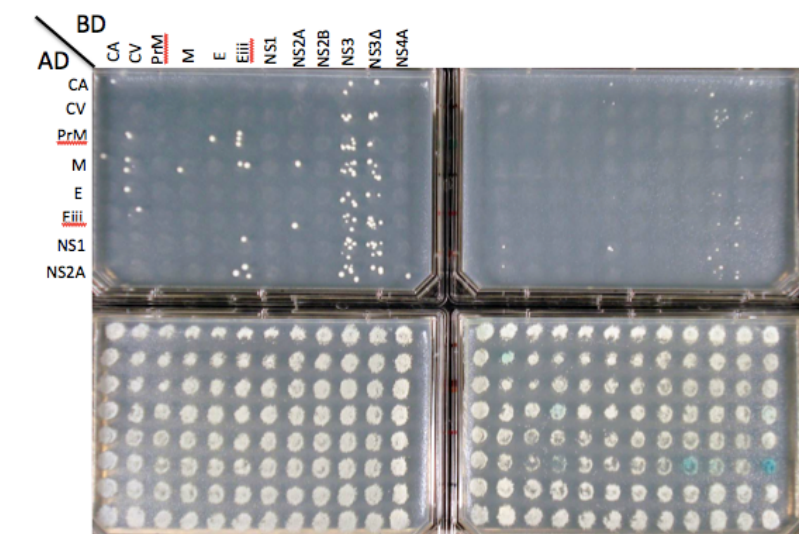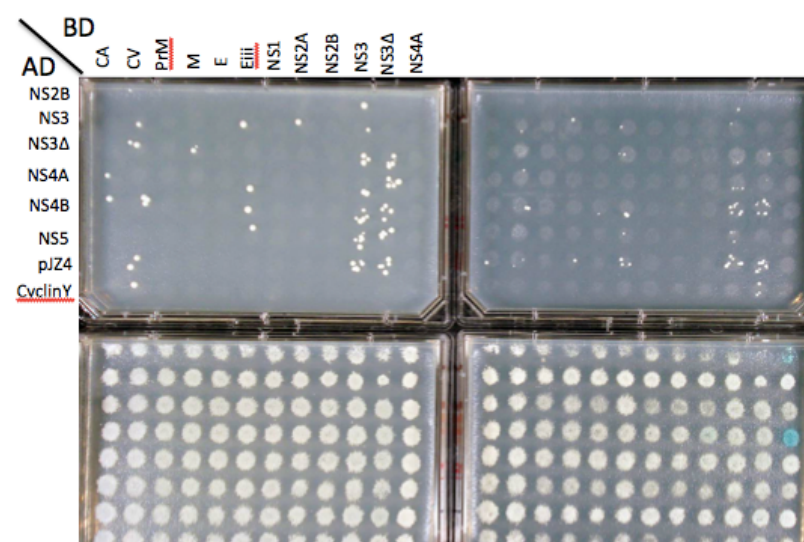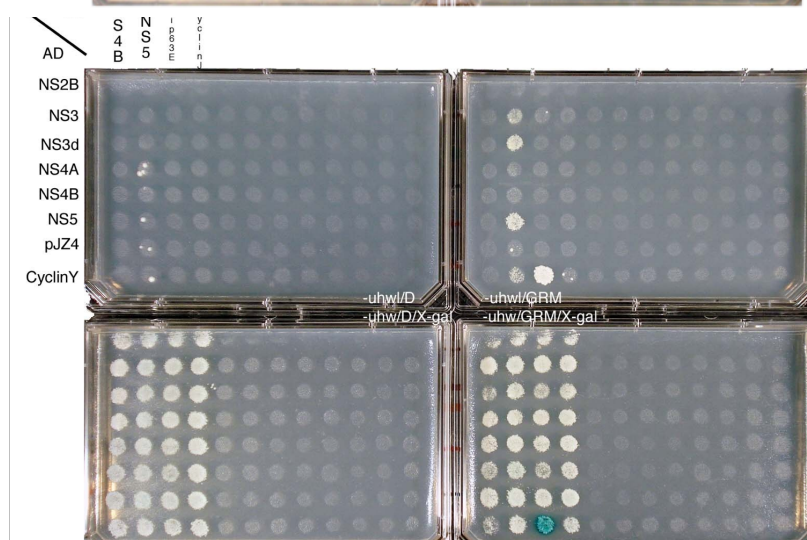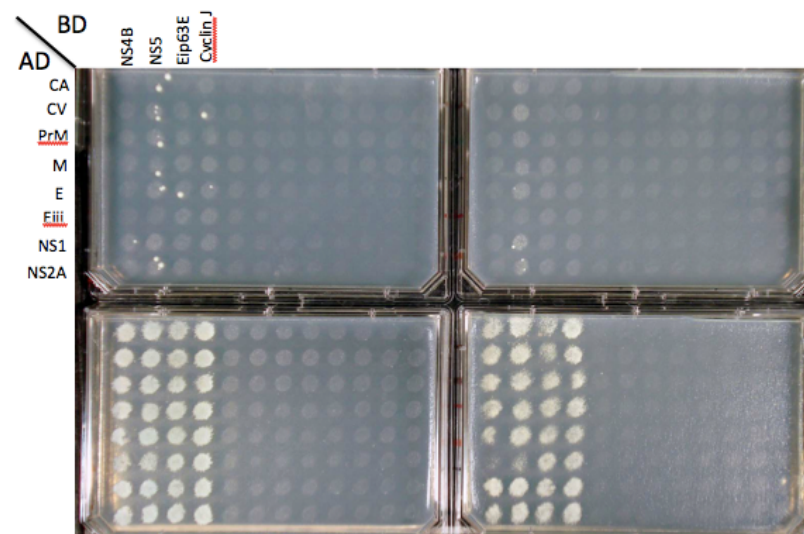

Supplement: Figure S1 — Intraviral protein-protein interactions. Interactions were identified by the galactose-dependent growth of diploid yeast expressing two dengue proteins. Each panel is a group of four indicator plates: Glucose complete minimal (CM) lacking leucine (–leucine) (top-left), Galactose CM –leucine (top-right), Glucose CM +X-gal (bottom-left) and Galactose CM +X-gal (bottom-left). An interaction is indicated by galactose-dependent growth on the plates lacking leucine (top two plates in each panel) or galactose-dependent blue colony color on the X-Gal plates (bottom two plates in each panel). Drosophila melanogaster Cyclin Y and Eip63E were used as a positive interaction control while D. melanogaster Cyclin Y and Cyclin J were used as a negative control. All media lack uracil, histidine, and tryptophan to select the two-hybrid plasmids. (PDF) [file pone.0053535.s012.pdf]

Fig. S2

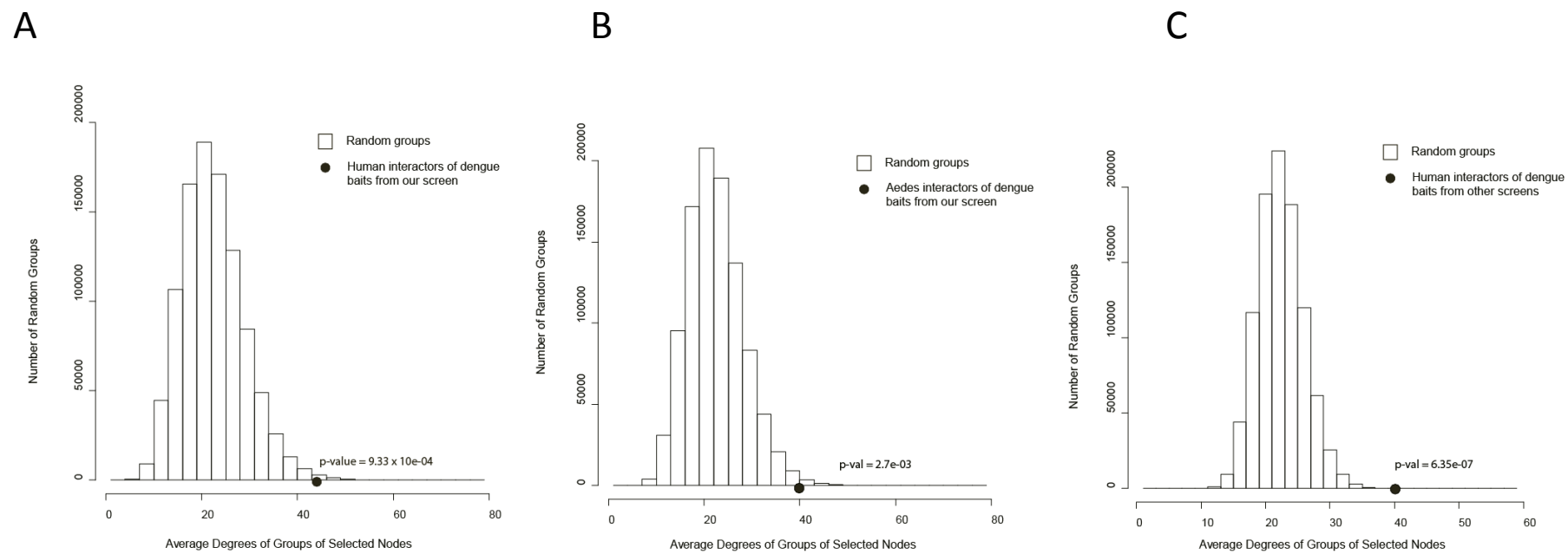

Supplement: Figure S2 — Dengue-interacting host proteins are enriched for hubs. Enrichment analyses for proteins with many interacting partners in three dengue-host interactomes: human interactors of dengue baits identified in this screen (A), human interactors of dengue baits identified in other screens (B), and Aedes aegypti interactors of dengue baits in this screen (C). (PDF) [file pone.0053535.s013.pdf]

Fig S3

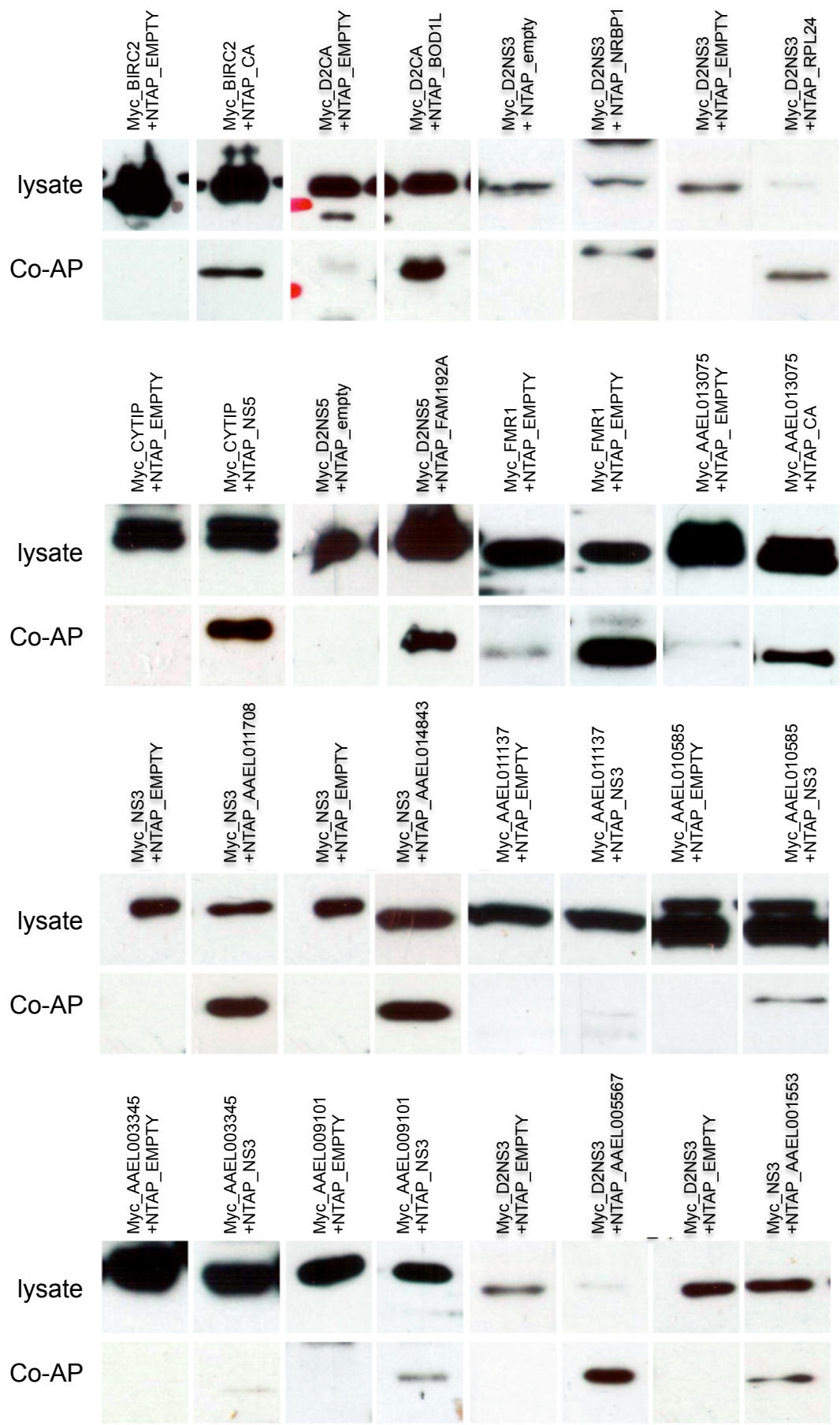

Fig S3, continued

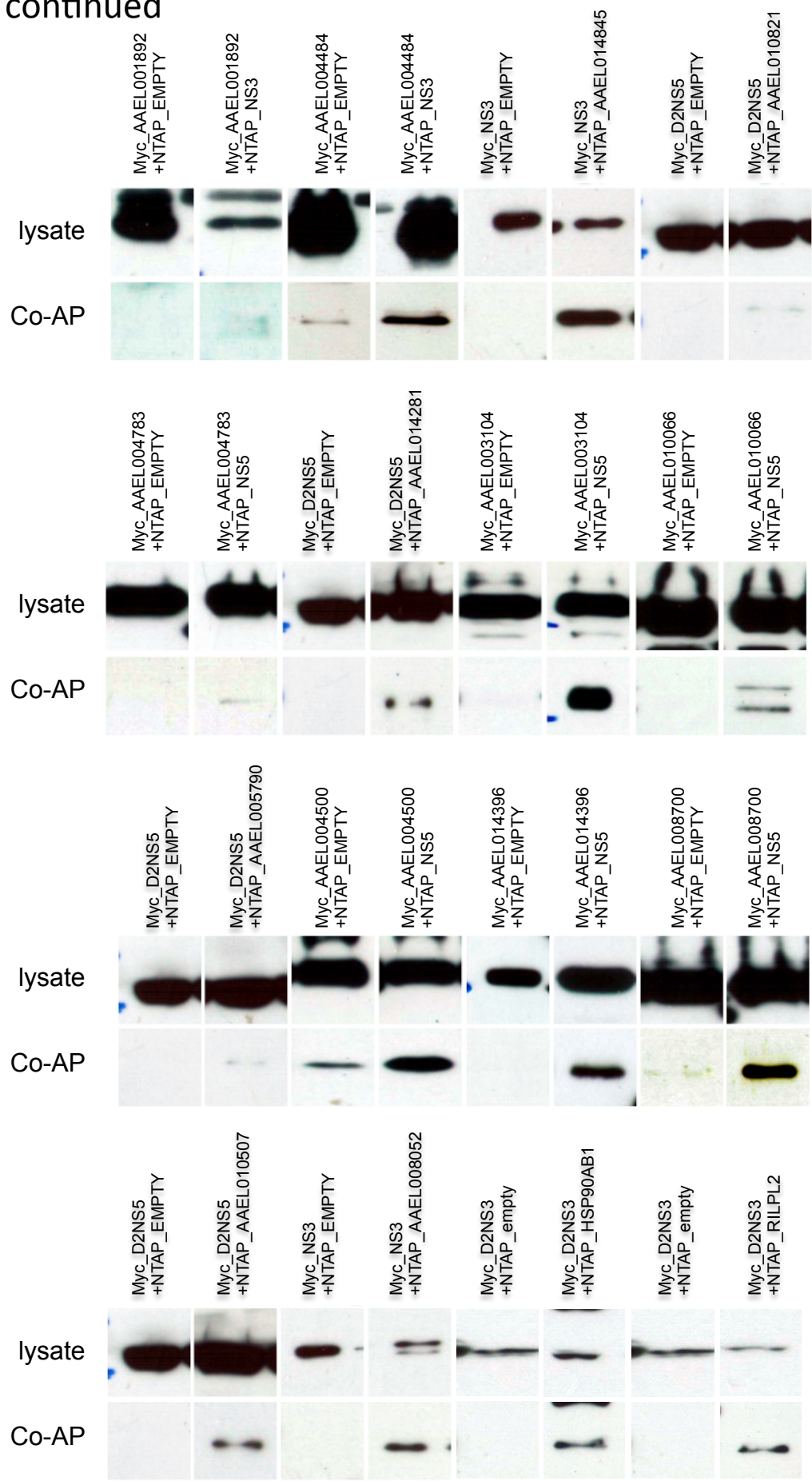

Supplement: Figure S3 — Co-AP assays for dengue-host protein interactions. Additional co-AP results that were not shown in Figure 3. Details are as described in Figure 3. (PDF) [file pone.0053535.s014.pdf]
